# Construction and Application of a Quantitative Perforation Erosion Model Based on Field Experiments

**DOI:** 10.3390/ma18112507

**Published:** 2025-05-26

**Authors:** Bo Wang, Huan Li, Enyu Zhang, Jinglong Ma, Zichen Shang, Xiongfei Liu

**Affiliations:** 1 Petroleum Institute, China University of Petroleum-Beijing at Karamay, Karamay 834000, China; wangbo@cupk.edu.cn (B.W.); lihuancupk@163.com (H.L.); 17599901216@163.com (E.Z.); 2024216884@st.cupk.edu.cn (J.M.); zc13663280176@163.com (Z.S.); 2 Research Institute of Unconventional Oil and Gas Science and Technology, China University of Petroleum-Beijing, Beijing 102249, China

**Keywords:** unconventional oil and gas, sand fracturing, perforation hole erosion, fracture propagation

## Abstract

Perforation erosion is one of the critical factors influencing the effectiveness of hydraulic fracturing and the productivity of oil and gas wells. This study developed a mathematical model for perforation erosion based on the field experimental data and theoretical analysis. This model comprehensively considers the effects of the rate of change in perforation diameter and the flow coefficient. Through field experiments, the values of the perforation diameter correlation coefficient (*α*) and the flow coefficient correlation coefficient (*β*) were determined. The wear behavior of perforations under high-pressure sand-carrying fluid conditions was thoroughly investigated, and the primary factors influencing perforation erosion were systematically analyzed. The results indicate that perforation erosion under high-pressure sand-carrying fluid conditions undergoes two distinct stages: the roundness erosion stage, characterized by a sharp pressure drop (greater than 30%) and the diameter erosion stage, marked by a gradual pressure decline (less than 5%), ultimately forming a trumpet-shaped perforation channel. The study further revealed that larger proppants cause significantly severe erosion than smaller proppants, resulting in 18.19% greater perforation diameter enlargement. In comparison tests, ceramic proppants produced 16.87% more diameter expansion than quartz sand under identical erosion conditions. Innovatively, this study proposes a “limited entry and temporary plugging” synergistic composite process. The timing of temporary plugging and the selection criteria for diverter size were clarified and optimized by determining the critical perforation friction for limited-entry failure based on inter-cluster stress differences. Field applications demonstrate that the optimized approach reduces erosion rates by 35–50%, improves fracture uniformity to over 80%, and increases single-well productivity by 18–25%. This research provides a quantitative basis and practical guidance for optimizing fracturing operation parameters, offering significant insights for enhancing the efficiency and productivity of hydraulic fracturing in oil and gas wells.

## 1. Introduction

The tight conglomerate reservoirs in the Mahu Sag of the Junggar Basin represent a critical replacement area for China’s onshore deep oil and gas resources. However, their development faces multiple challenges, including significant burial depth (>4500 m), firm stress heterogeneity (variation coefficient >0.65), and complex natural fracture systems [1,2,3]. The multi-cluster fracturing technology in horizontal wells, achieved through optimized cluster spacing (5–10 m) and cluster density (6–12 clusters per stage), has demonstrated significant advantages in achieving volumetric stimulation of reservoirs [4,5]. Nevertheless, with the large-scale application of “three-high” fracturing techniques (high pumping pressure >90 MPa, high flow rate >12 m^3^/min, and high sand ratio >30%), perforation holes undergo morphological distortion under the continuous erosion of high-pressure sand-carrying fluids. This leads to abnormal near-wellbore friction fluctuations, severely restricting the uniform propagation of fractures [6,7].

Current research on perforation erosion primarily follows three directions:

(1) Numerical Simulation: Liu Yanshu et al. [8] revealed the synergistic effect of flow rate and sand concentration on erosion rates using the discrete element method, but the characterization of multi-physical field coupling remains challenging. Huang et al. [9] developed a multi-field coupled fracturing model integrating stress–seepage–fracture–erosion mechanisms using the continuous-discontinuous element method (CDEM). Through benchmark case studies, they investigated the impact of perforation erosion on multi-fracture propagation. They analyzed the influence of flow rate and perforation parameters on perforation erosion and fracture extension. The results demonstrate that perforation erosion non-uniformly enlarges perforation diameter and increases the flow coefficient, leading to redistributed flow among multiple fractures and exacerbating uneven fracture propagation. Hu et al. [10] established a 3D CFD wellbore model for single- and dual-cluster fractures. By incorporating erosion effects, they studied proppant transport behavior in slickwater fracturing. The findings reveal that proppant distribution is significantly affected by perforation erosion location, erosion angle, erosion severity, and fluid injection velocity.

(2) Experimental Studies: The high-speed two-phase flow erosion system developed by Li Zhen’s team [11] can simulate the bridging process of temporary diverters. However, the centimeter-scale perforations and static stress conditions result in scale effect errors exceeding 40%. Wu et al. [12] developed a surface-perforation erosion simulation system to quantitatively investigate the erosion extent of perforations during sand-carrying fracturing. They studied the erosive effects of sand-laden fluids on individual perforations under varying operational conditions, clarifying the changes in perforation geometry and post-erosion perforation friction in real-world fracturing scenarios. Wang Bo et al. [13] constructed a high-pressure perforation plugging simulation system and prepared five perforation models with distinct geometries. Their research focused on the influence of temporary plugging ball diameter, bridging particle concentration, filler particle concentration, pumping rate, and perforation shape on the effectiveness of perforation sealing.

(3) Field Monitoring Techniques: Zang Chuanzhen et al. [14] employed distributed optical fiber monitoring to observe that perforation erosion causes over 30% of perforation clusters to lose fluid intake capacity, yet quantitative description methods are lacking. The limitations of these approaches are evident: numerical models oversimplify boundary conditions, physical experiments struggle to replicate actual scale effects, and field monitoring lacks dynamic process analysis [15,16]. Wheaton et al. [17] monitored multi-cluster fracturing operations in the Eagle Ford Shale using DAS (distributed acoustic sensing) and DTS (distributed temperature sensing) technologies. The results revealed that heel-side perforations dominated fluid intake. Under the exact total perforation count, increasing the number of perforation clusters led to a gradual rise in the proportion of ineffective clusters. Additionally, due to stress shadow effects, minimal cluster spacing severely inhibited the initiation of specific perforation clusters. Cramer et al. [18] employed DAS, treatment pressure analysis, and perforation imaging to monitor fluid and proppant distribution during limited-entry fracturing. They qualitatively evaluated fluid intake per cluster by comparing perforation diameters before and after fracturing. The results indicated that wellbore pressure dropped after proppant addition, with some clusters showing no fluid intake, while heel-side perforations exhibited more pronounced erosion. Roberts et al. [19] utilized downhole video imaging (e.g., Eagle Eye) to collect detailed perforation size data from over 6000 clusters and 600 stages. By analyzing erosion patterns, they identified the impacts of stage length, cluster count, cluster spacing, perforation density, charge type, and stage number on proppant distribution. The study confirmed that heel-side perforations experienced more severe erosion than toe-side perforations, demonstrating non-uniform proppant distribution along the stage, with heel clusters receiving a higher proppant volume.

This study innovatively constructs a tripartite research methodology integrating field experiments, theoretical modeling, and engineering control. A field-scale perforation erosion simulation device was developed, enabling dynamic erosion observation of full-scale perforations (Φ8–12 mm) under realistic in situ stress conditions. An improved dynamic prediction model for perforation erosion based on the Long model was established, incorporating a dynamic friction correction factor to overcome the limitations of traditional models in characterizing inter-cluster interference effects. A temporary plugging parameter optimization method was proposed, using “inter-cluster stress difference” as the core criterion. Providing a new technical pathway for the efficient development of Mahu reservoirs.

## 2. Field-Scale Perforation Erosion Experiments

During hydraulic fracturing operations in the Mahu conglomerate reservoirs, the pumping rate, proppant concentration, and injection pressure reach up to 14 m^3^/min, 420 kg/m^3^, and 90 MPa, respectively. As the sand-laden fluid passes through the perforations, erosion of the perforation holes is inevitable. This erosion compromises the effectiveness of limited-entry fracturing. It increases the difficulty and uncertainty of temporary perforation plugging, leading to uneven propagation of multiple fracture clusters within a single stage of horizontal well fracturing. Current laboratory studies often employ low pumping rates, low pressures, and small proppant volumes, significantly different from actual field fracturing conditions. To address this gap, this study utilizes a field-scale, high-intensity perforation erosion simulation system, incorporating fracturing trucks and actual downhole casings, achieving perforation flow velocities of up to 200 m/s and injection pressures of up to 105 MPa. This research delves into the dynamic characteristics and influencing mechanisms of perforation erosion by replicating actual field conditions.

### 2.1. Design of the Field-Scale High-Intensity Perforation Erosion System

**Figure 1:**
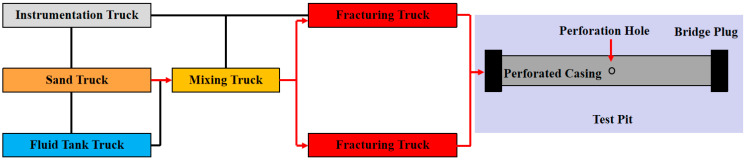
Schematic diagram of the perforation erosion evaluation system.

**Figure 2:**
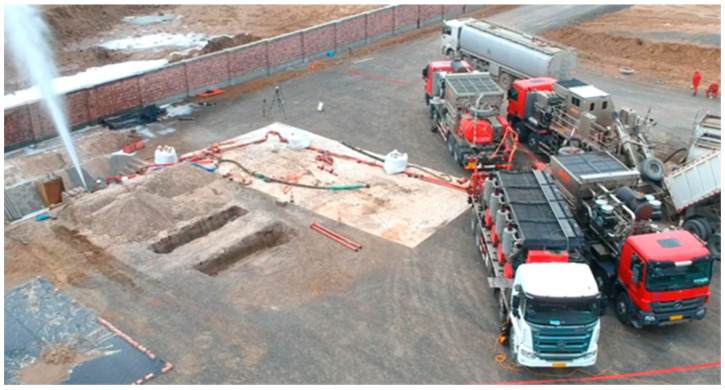
Field-scale perforation erosion evaluation system.

The field-scale perforation erosion experiments aim to investigate the impact of sand-carrying fluids on perforation erosion under actual fracturing conditions (high rate, high pressure, and high sand ratio). As illustrated in Figure 1 and Figure 2, the system comprises fracturing trucks, liquid storage trucks, sand transport trucks, mixing trucks, instrumentation trucks, high-pressure pipelines, and perforated casings. The fracturing truck assembly enables maximum fluid velocities of 200 m/s and injection pressures of up to 100 MPa. Liquid storage trucks continuously supply fracturing fluid, each capable of storing 30 m^3^ of fluid, ensuring operational flexibility and on-demand fluid replenishment. Sand transport trucks are primarily used for proppant storage and supply, with each tank capable of holding 20 m^3^ of proppant. Mixing trucks are responsible for blending, agitating, and transporting the proppant. The instrumentation truck provides real-time control and monitoring of pressure, flow rate, fluid density, and hydraulic power, allowing for dynamic adjustment of pumping parameters to ensure experimental safety. The high-pressure manifold serves as the main injection channel for the fracturing fluid, with a maximum pressure rating of 110.0 MPa. A specially designed fluid storage tank is also used for fracturing fluid storage and flowback.

A 1 m long casing section, cut from the fracturing casing used in the Mahu conglomerate reservoirs, is prepared for the experiments. The TP125V steel casing has a pressure rating of up to 90.70 MPa, with an outer diameter of 139.7 mm and a wall thickness of 10.54 mm. One end the casing is threaded to connect to the high-pressure manifold, while the other is sealed by welding. A 10 mm diameter perforation hole is drilled in the middle of the casing section using a diamond drill bit.

### 2.2. Experimental Methods and Procedures

The perforated casing was fixed in the experimental pool, connected to high-pressure pipelines, and prepared with fracturing fluid and proppant. Pressure, sealing, and safety tests were conducted before proceeding with the experiments according to the design outlined in Table 1.

Experiment 1: High-viscosity fracturing fluid was used to study the effect of fluid viscosity on the erosion coefficient.

Experiment 4: 20/40 mesh quartz sand was employed to investigate the influence of proppant size on the erosion coefficient.

Experiment 2: 40/70 mesh ceramic proppant was used to examine the impact of proppant type on the erosion coefficient.

Experiment 3: Different sand concentrations were tested to analyze the effect of proppant concentration on perforation erosion.

## 3. Perforation Erosion Model

### 3.1. Construction of the Perforation Erosion Prediction Model

When high-pressure fluid flows through the perforations in oil and gas wells, the surrounding material is subjected to fluid impact forces, leading to gradual erosion and wear. This increases perforation size, adversely affecting well productivity, flow characteristics, and wellbore integrity. The Long model [20] is a mathematical model that describes and predicts perforation erosion caused by the flow of liquids or gases through perforations, typically employed in well-completion operations. The model focuses on the changes in perforation size, flow resistance, and material loss at the perforation edges due to the impact and shear forces exerted by high-pressure, high-velocity fluids.

The core of the Long model lies in its mathematical formulation, which describes the geometric changes in perforations under fluid erosion, particularly the enlargement of the perforation diameter and the variation in the flow coefficient. The model incorporates factors such as fluid velocity, particle concentration, fluid density, and the wear resistance of the perforation material. The key equations of the Long model are as follows:

1. Perforation diameter change equation [20]:

(1) $$\frac{dD}{dt}=\alpha C\nu^{2}$$

where *D* is the perforation diameter (m), *t* is time (s), the parameter *α* is the perforation diameter erosion coefficient, which characterizes the influence of proppant kinetic energy, represented by the product of sand concentration and the square of flow velocity, on the growth rate of perforation diameter. Its unit is (m^2^·s/kg). *C* is the sand concentration (kg/m^3^), and *v* is the fluid velocity (m/s).

2. Flow coefficient change equation [20]:

(2) $$\frac{dC_{d}}{dt}={\beta C}^{\nu^{2}}(1-\frac{C_{d}}{C_{d}^{max}})$$

where *C_d_* is the flow coefficient (dimensionless), the parameter *β* is the flow coefficient erosion coefficient, reflecting the effect of kinetic energy on the smoothing of perforation edges, that is, its contribution to the growth rate of the flow coefficient *C_d_*. Its unit is (m·s/kg). *C_max_* is the maximum flow coefficient, *C* is the sand concentration (kg/m^3^), and *v* is the fluid velocity (m/s).

3. Fluid Velocity Expression:

(3) $$\nu =\frac{4q}{n\pi D^{2}}$$

where *v* is the fluid velocity (m/s), *Q* is the flow rate (m^3^/s), *n* is the number of perforations (dimensionless), and *D* is the perforation diameter (m).

4. Pressure drop equation:

(4) $$P_{r}=\frac{8\rho }{\pi^{2}C_{d}^{2}D^{4}}{\left(\frac{q}{n}\right)}^{2}$$

where *P_r_* is the pressure drop (Pa), ρ is the fluid density (kg/m^3^), *Q* is the flow rate (m^3^/s), *n* is the number of perforations (dimensionless), *C_d_* is the flow coefficient (dimensionless), and *D* is the perforation diameter (m).

### 3.2. Calculation of α and β Based on Experimental Data

Solid particles impact the perforation edges at high speeds when high-velocity fluid flows through perforations. Due to the complex flow regime, different regions of the perforation edges experience varying forces, leading to localized mechanical wear. As particles continuously impact the edges, the material gradually erodes, causing the perforation edges to become irregular and uneven. This phenomenon, known as roundness erosion, results in the perforation shape deviating from its original circular form, becoming asymmetric or rough. Additionally, diameter erosion occurs as the overall perforation diameter increases due to prolonged particle impact and continuous wear on the perforation walls. Over time, the perforation diameter significantly enlarges.

**Figure 3:**
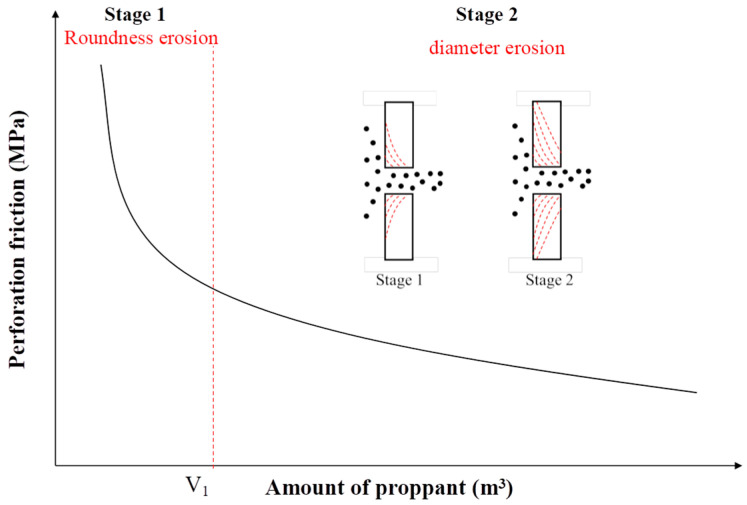
Roundness erosion stage and diameter erosion stage during sand-laden erosion [21].

In summary, roundness erosion primarily affects the shape of the perforation edges, reducing their smoothness and causing irregularity. In contrast, diameter erosion refers to the overall increase in perforation size due to erosion. The changes in perforation shape and size directly influence the flow characteristics of fluids passing through the perforations. As shown in Figure 3, the roundness erosion stage is characterized by a sharp pressure drop (>30%), while the diameter erosion stage exhibits a gradual pressure decline (<5%). The process involves roundness loss (40–55% reduction in roundness) and diameter expansion (1.8–2.5 times the original diameter). A trumpet-shaped perforation with an inner diameter more than twice the outer diameter is formed. Specifically, perforation expansion reduces pressure loss and improves fluid flow efficiency, while irregular perforation shapes may cause localized flow disturbances, increase flow resistance, and mitigate fracturing fluid performance.

Equation (1) characterizes diameter erosion, where the perforation diameter changes as proppant particles are continuously injected. Equation (1) is only applicable to the “diameter erosion stage”; during the “roundness erosion stage”, the perforation evolution should be characterized by the change in the flow coefficient *C_d_*. Equation (2) characterizes roundness erosion, where the perforation shape changes significantly as proppant particles erode the edges, leading to variations in the flow coefficient. To solve for *α* and *β*, Equation (3) is first substituted into Equation (1), and the resulting expression is integrated to obtain:

(5) $$D^{5}=\frac{80\alpha Cq^{2}}{n^{2}\pi^{2}}t+D_{0}^{5}$$

Substituting Equation (5) into Equation (4), the solution reveals that roundness erosion occurs first, followed by diameter erosion. Therefore, during diameter erosion, the flow coefficient reaches its maximum value:

(6) $$P_{r}^{-1.25}=\alpha (\frac{n^{2}\pi^{2}}{8\rho q^{2}}){\left(C_{d}^{max}\right)}^{2.5}\frac{80Cq^{2}}{n^{2}\pi^{2}}t+{\;(\frac{n^{2}\pi^{2}}{8\rho q^{2}})}^{1.25}\;{\left(C_{d}^{max}\right)}^{2.5}D_{0}^{5}$$

**Figure 4:**
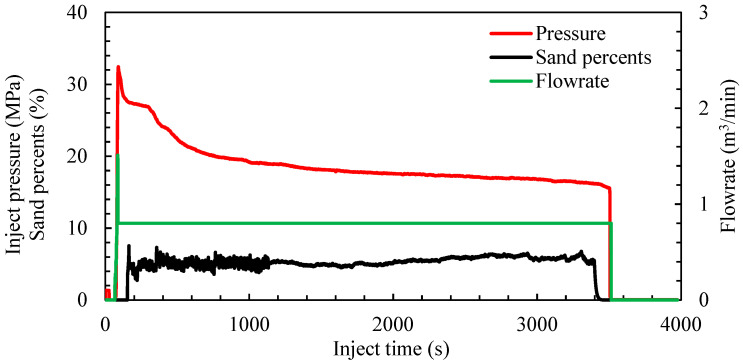
Test 3 operational curve.

**Figure 5:**
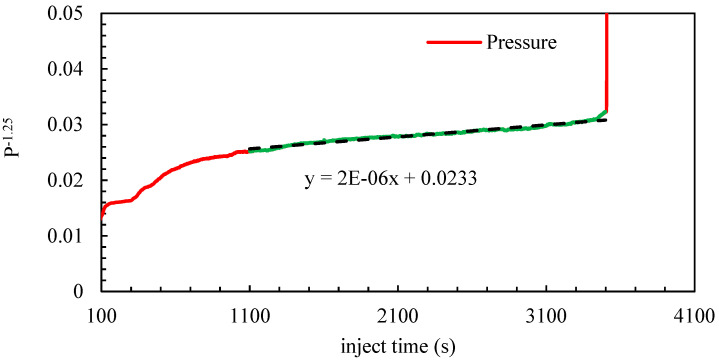
The diameter erosion stage *P*^−1.25^-*t* curve.

**Figure 6:**
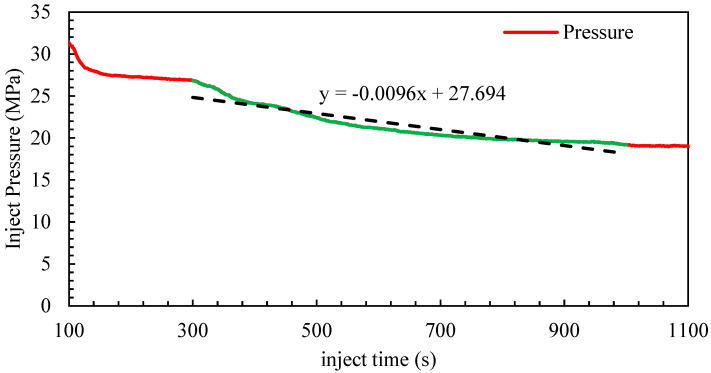
The roundness erosion stage *P*-*t* curve.

As shown in Table 1, the experimental parameters for Test 3 include a flow rate of 0.9 m^3^/min, 40/70 mesh quartz sand, and a sand concentration of 90 kg/m^3^. By dividing the treatment curve of Test 3 (as shown in Figure 4) into the roundness erosion stage and the diameter erosion stage, the diameter erosion coefficient *α* was determined. Figure 5 and Figure 6 correspond to two different stages of the erosion process. Due to the shift in dominant mechanisms, we adopted different forms of pressure, such as the vertical axis in each figure. Figure 5 corresponds to the “diameter erosion stage”, where *C_d_* has nearly reached its maximum value, and the expansion of the orifice diameter *D* becomes the dominant factor. From the model, pressure follows *P_r_*∝1/*D*^4^, and combining this with the geometric relationship *D*^5^∝*t*, it follows that *P_r_*^−1.25^∝*t*. Therefore, we use *P_r_*^−1.25^ as the vertical axis to linearize the relation for regression and extract parameter *α*.

Figure 6 analyzes the “roundness erosion stage”, during which the orifice diameter remains nearly constant. At this stage, the primary change arises from increased discharge coefficient *C_d_*. According to the model, pressure satisfies the relationship *P_r_*∝1/*C_d_*^2^. Therefore, the original pressure *P* is used as the vertical axis. By taking the time derivative of Equation (4), the regression of parameter *β* is performed. Considering that both the discharge coefficient *C_d_* and the perforation diameter *D* change during the erosion process, Equations (1) and (2) are also incorporated, yielding:

(7) $$\frac{dP_{r}}{\mathrm{dt}}=\;-\frac{16q^{2}C\nu^{2}\rho }{n^{2}\pi^{2}C_{d}^{2}D^{4}}\;\left[\beta (\frac{1}{C_{d}}-\frac{1}{C_{d}^{max}})+\frac{2\alpha }{D}\right]$$

when *t* = 0, *C_d_* = *C_d_*_0_ and *D* = *D*_0_, leading to:

(8) $$\frac{dP_{r}}{\mathrm{dt}}=\;-P_{r0}\frac{32Cq^{2}}{n^{2}\pi^{2}D_{0}^{4}}\;\left[\beta (\frac{1}{C_{d0}}-\frac{1}{C_{d}^{max}})+\frac{2\alpha }{D}\right]$$

As shown in Figure 6, the flow coefficient correlation coefficient *β* is calculated using the above equations.

## 4. Quantitative Characteristics of Perforation Erosion

### 4.1. Perforation Erosion Under Different Fluid Viscosities

**Figure 7:**
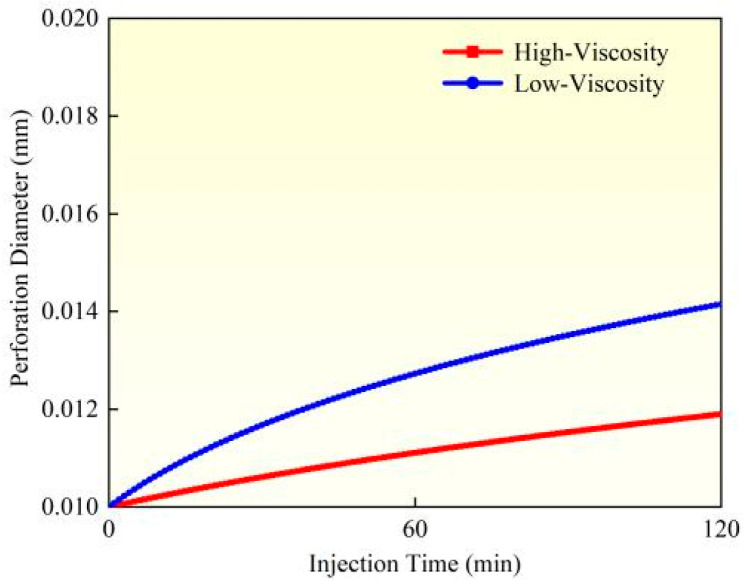
Hole diameter dynamic change.

**Figure 8:**
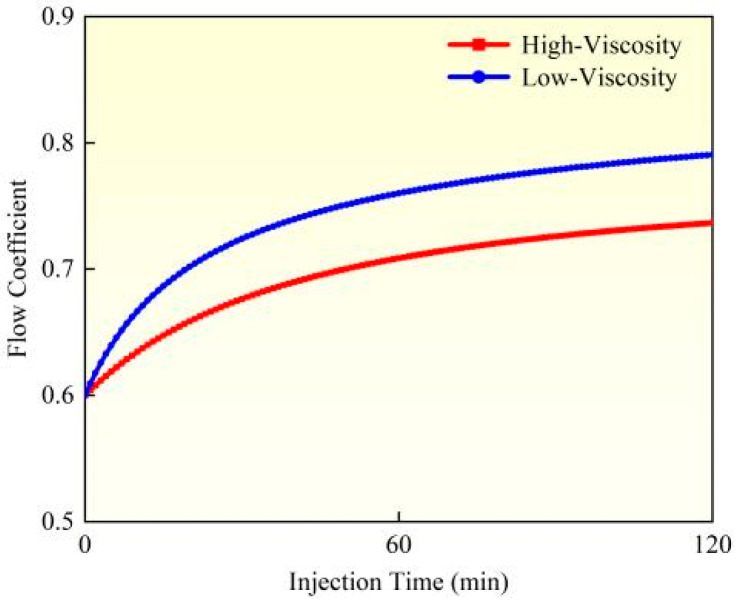
Flow coefficient dynamic changes.

**Figure 9:**
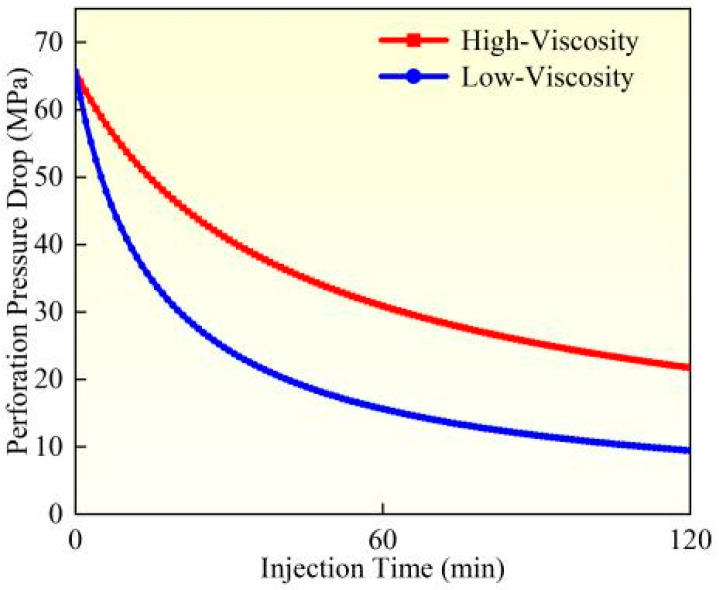
Hole pressure drop dynamic changes.

While considering proppant suspension characteristics, the influence of fracturing fluid viscosity on perforation erosion exhibits significant variations. A comparative study between Experiment 1 and Experiment 3 was conducted to analyze the erosion patterns under different fluid viscosity conditions. Based on the perforation erosion model, this research evaluated the dynamic evolution of perforation diameter, flow coefficient, and pressure drop during erosion (as illustrated in Figure 7, Figure 8 and Figure 9). High-viscosity fracturing fluids (e.g., crosslinked gels) demonstrate enhanced proppant transport capacity, effectively reducing particle settling and mitigating localized impact erosion along perforation edges. Furthermore, the lower flow velocity of high-viscosity fluids under equivalent pumping rates diminishes shear stress on perforation walls, alleviating material wear. Experimental data reveal that when fluid viscosity decreases from high (60 mPa·s) to low (10 mPa·s), calculated based on comparative analysis of model results, the perforation diameter increases by 18.84%, the flow coefficient rises by 7.32%, and the perforation pressure drops by 21.99%. In contrast, low-viscosity fluids (e.g., slickwater) exhibit inferior suspension performance, leading to proppant settling and accumulation near the perforation base. This results in elevated local particle concentration and intensified impact erosion. The higher flow velocity of low-viscosity fluids also amplifies turbulence and shear forces, directly accelerating the mechanical wear of perforation edges. Experimental observations confirm more severe erosion intensity and the potential formation of trumpet-shaped perforations. To address these issues, field applications should prioritize high-viscosity fracturing fluids (≥50 cP) to optimize the balance between proppant transport and erosion control. In summary, fluid viscosity governs erosion behavior through dual mechanisms: proppant distribution and hydrodynamic shear. High-viscosity fluids suppress erosion via uniform particle suspension and reduced shear, whereas low-viscosity fluids exacerbate erosion through particle settling and enhanced shear stress.

### 4.2. Perforation Erosion Under Different Proppant Types

**Figure 10:**
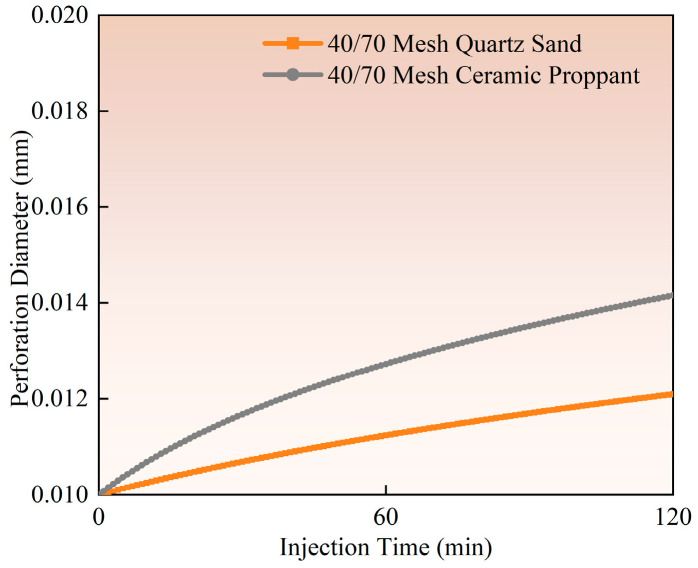
Hole diameter dynamic change.

**Figure 11:**
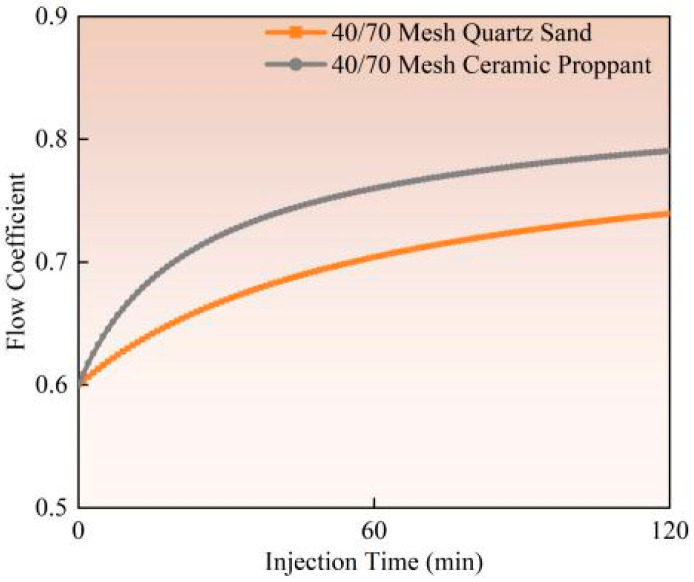
Flow coefficient dynamic changes.

**Figure 12:**
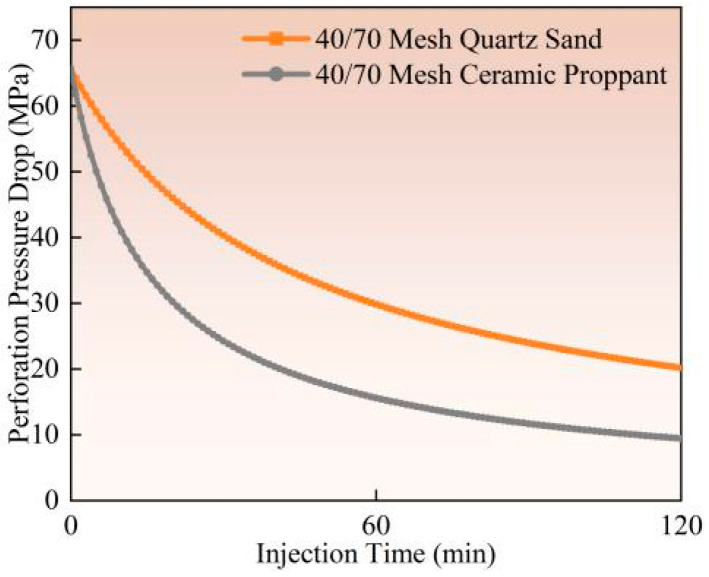
Hole pressure drop dynamic changes.

The influence of proppant type on perforation erosion exhibits significant variations, as demonstrated by experimental studies (Experiment 1 vs. Experiment 2) and field observation data. Ceramic proppants show markedly higher erosion levels than quartz sand (as illustrated in Figure 10, Figure 11 and Figure 12). Calculated based on comparative analysis of model results, with a 120 min pumping operation resulting in 16.87% and 6.91% increases in perforation diameter and flow coefficient, respectively, along with a 19.04% reduction in perforation pressure drop. Ceramic proppants’ superior compressive and shear strength leads to more severe material erosion under identical pumping conditions. In contrast, quartz sand’s lower mechanical strength causes particle fragmentation upon contact with casing surfaces, paradoxically reducing its cumulative erosive effect. Field-scale tests reveal that ceramic proppants form more pronounced trumpet-shaped perforations with inner diameters exceeding twice the original size, while quartz sand produces relatively uniform diameter enlargements. This divergence stems from ceramic particles maintaining structural integrity during high-velocity impacts, enabling sustained erosive action, whereas quartz sand dissipates erosive energy through particle breakage. Based on these findings, quartz sand proppants are recommended for field applications to mitigate the adverse effects of perforation erosion on pipeline materials and operational environments.

### 4.3. Perforation Erosion Under Different Proppant Sizes

**Figure 13:**
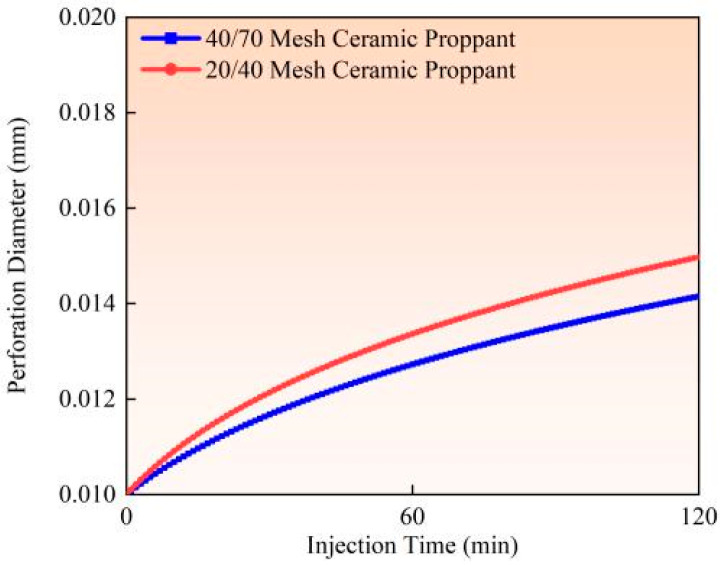
Hole diameter dynamic change.

**Figure 14:**
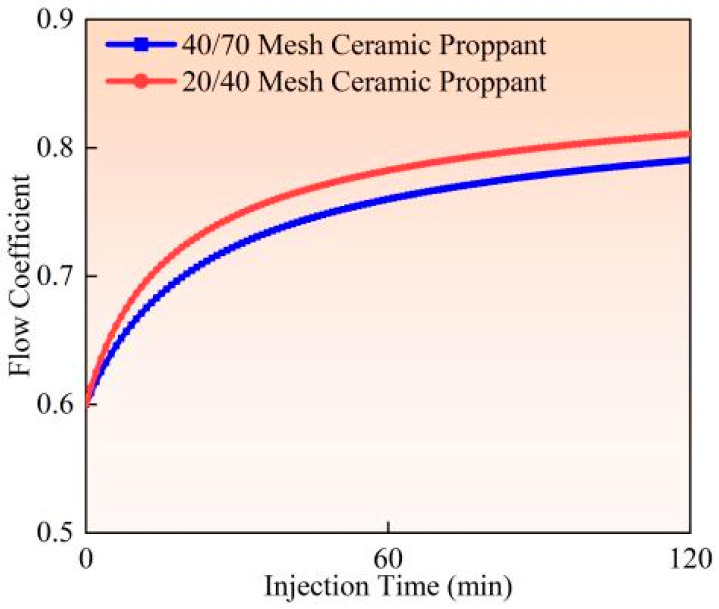
Flow coefficient dynamic changes.

**Figure 15:**
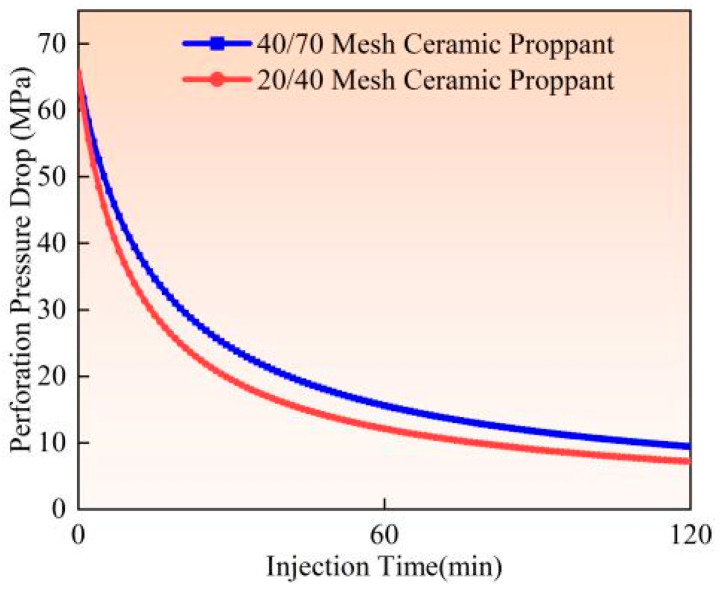
Hole pressure drop dynamic changes.

Experimental studies and field observations (Experiments 2 and 3) demonstrate that under identical pumping conditions (flow rate 0.9 m^3^/min, proppant concentration 90 kg/m^3^), larger-sized proppants (20/40 mesh) induce more severe perforation erosion than smaller-sized proppants (40/70 mesh) (as illustrated in Figure 13, Figure 14 and Figure 15). Calculated based on comparative analysis of model results, after 120 min of pumping, the larger proppants caused an 18.19% increase in perforation diameter, a 5.47% improvement in flow coefficient, and an 8.34% reduction in perforation pressure drop. This phenomenon primarily results from the greater contact area between larger proppants and perforation surfaces, combined with their higher kinetic energy per particle and more concentrated impact forces. Full-scale field tests further confirm that 20/40 mesh proppants create more substantial erosion on perforation surfaces, leading to trumpet-shaped deformation of the perforations. The underlying mechanical mechanism lies in the superior momentum conservation of larger particles during high-velocity flow, which generates more intense point impacts on perforation edges. In contrast, although smaller particles are more numerous, their impact energy is more dispersed, and their contact area with surfaces is comparatively reduced. The engineering practice commonly adopts a “small-to-large” proppant injection strategy; finer proppants (40/70 mesh) are preferentially injected to penetrate deeper into fracture networks. In comparison, coarser proppants (20/40 mesh) are subsequently placed near the fracture entrance to establish high-conductivity proppant packs. Field applications should prioritize smaller mesh proppants to mitigate perforation erosion while enhancing proppant placement efficiency.

### 4.4. Perforation Erosion Under Different Sand Concentrations

**Figure 16:**
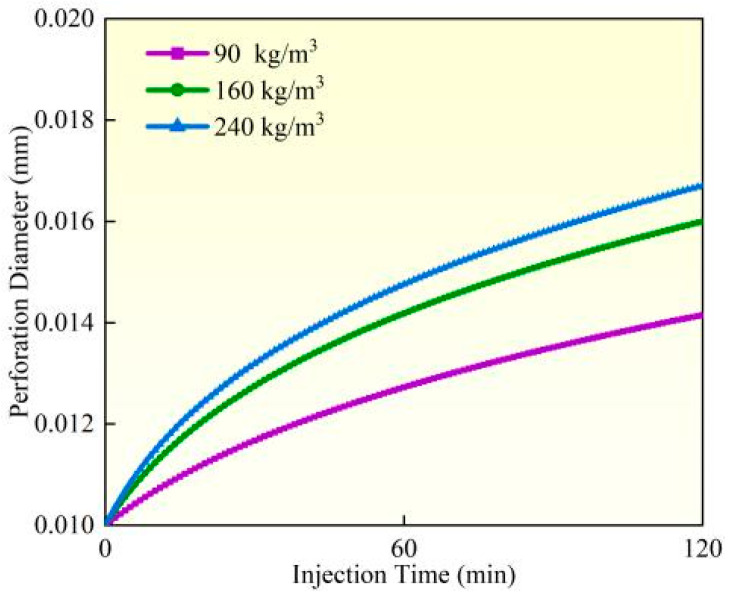
Hole diameter dynamic change.

**Figure 17:**
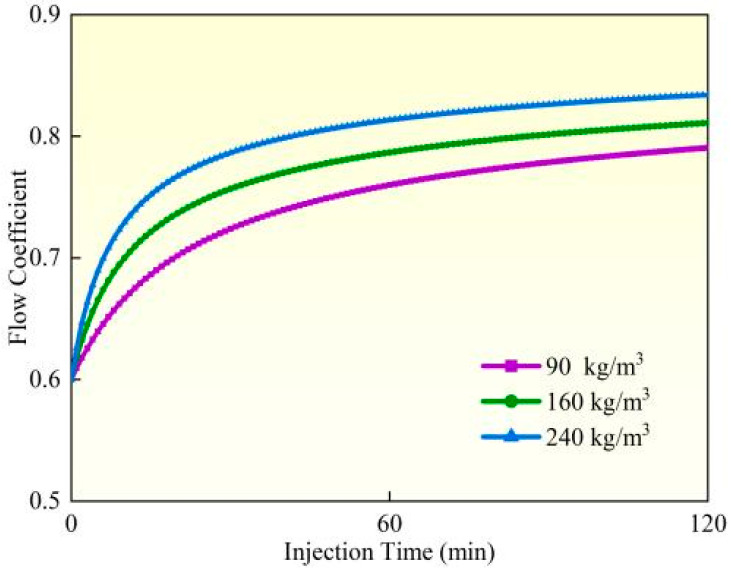
Flow coefficient dynamic changes.

**Figure 18:**
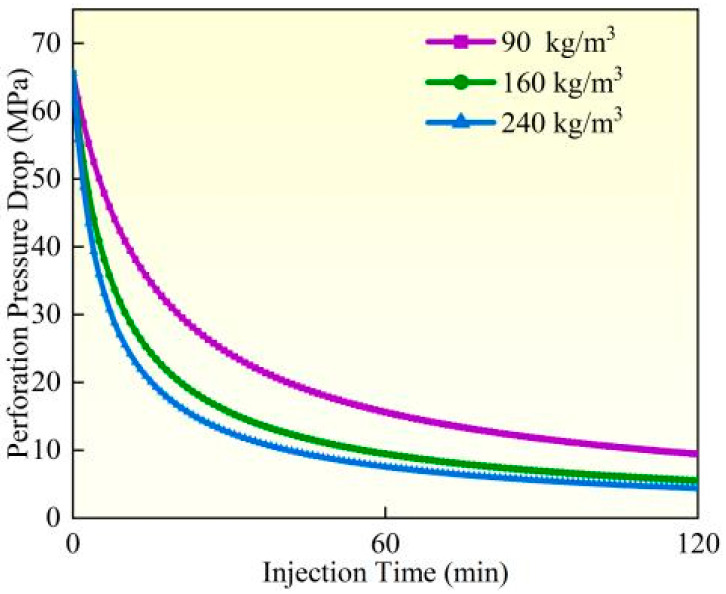
Hole pressure drop dynamic changes.

Experimental studies and field observations (Experiment 3) demonstrate that under identical pumping conditions, perforation erosion intensifies significantly as proppant concentration increases from 90 kg/m^3^ to 240 kg/m^3^ (Figure 16, Figure 17 and Figure 18). Calculated based on comparative analysis of model results, after 120 min of pumping, the perforation diameter and flow coefficient increased by 18.19% and 5.47%, respectively, while the perforation pressure drop decreased by 8.34%. Under high proppant concentrations, the perforation surfaces exhibit more severe localized erosion characteristics, forming erosion pits of varying depths and distinct trumpet-shaped deformation, with the inner diameter expanding up to twice its original size. The underlying mechanism reveals that as proppant concentration increases, the number of particles impacting the perforation wall per unit of time grows linearly, while enhanced inter-particle interactions leading to substantially more significant cumulative erosion effects. Based on these findings, field operations should employ optimized proppant concentration gradient designs (e.g., using lower concentrations initially and gradually increasing during middle-to-late stages) to balance erosion effects with proppant placement requirements while simultaneously implementing real-time pressure monitoring to prevent screen outs.

## 5. Application Case Study of Perforation Erosion Model

### 5.1. Application Methodology of the Perforation Erosion Model

Limited entry and temporary plugging fracturing techniques are commonly employed to promote simultaneous initiation and propagation of multiple fracture clusters in horizontal wells [22,23,24]. The core principle of limited-entry fracturing is to increase the perforation friction within the horizontal wellbore, thereby restricting the fluid intake capacity of each cluster and ensuring simultaneous fracture initiation and propagation [25]. However, field-distributed fiber optic monitoring [26] and downhole camera observations reveal that fracture propagation is often uneven, and the continuous erosion of perforations by sand-laden fluids leads to the failure of limited-entry techniques. This underscores the significant impact of perforation erosion on the effectiveness of limited-entry fracturing.

This study constructs a perforation erosion model that accounts for the effects of erosion on fracture propagation. A “limited entry + temporary plugging” composite process is proposed to achieve balanced reservoir stimulation. As the number of perforations decreases, perforation friction increases, overcoming inter-cluster stress differences and promoting uniform fluid intake and fracture propagation. However, during fracturing operations, the injection of large volumes of quartz sand causes perforation erosion, leading to the deformation and enlargement of the perforations. This reduces perforation friction, rendering the limited-entry technique ineffective. To address this issue, a temporary plugging process is introduced, utilizing degradable fiber-based diverters to seal dominant perforations, creating localized blockages dynamically. This redistributes fluid flow within the wellbore, forcing fluid into unsealed perforations and promoting the extension of underdeveloped fractures. The diverters, made of degradable fiber materials, exhibit high bridging capability, rapidly forming effective seals and naturally dissolving post-operation to avoid wellbore or fracture blockages.

### 5.2. Field Application and Analysis

Well A, a typical horizontal well in the Mahu oilfield, was selected for this study. Located in the Xia 72 fault block of the Mahu 131 well area, the target formation is the Triassic Baikouquan Formation (T_1_b_3_^2^). The well has a vertical depth of 2.721.76 m, a measured depth of 4850 m, and a horizontal section length of 1940 m. The well employs a dense-cluster design for volumetric fracturing, utilizing plug-and-perf staged fracturing for large-scale stimulation, with consideration given to perforation erosion effects. Temporary plugging was applied during the fracturing of stages 7–15 and 17, using fiber-based diverters to seal perforations. Table 2 shows that three different perforation densities per cluster (3, 5, and 8 perforations per cluster) were tested for limited-entry fracturing.

A comparative test was conducted on Well A, stages 7–15 (9 fracturing stages). Each stage was designed with 6 clusters, and each cluster was created with a proppant volume of 25 m^3^, resulting in an average proppant intensity of 1.2 m^3^/m. To eliminate spatial randomness, stages 7, 10, and 13 were designed with three perforations per cluster, stages 8, 11, and 14 with five perforations per cluster, and stages 9, 12, and 15 with eight perforations per cluster. The perforation erosion parameters, calculated from field tests in the Mahu area, are *α* = 9.85 × 10^−14^ and *β* = 9.48 × 10^−10^ and were used to construct the perforation erosion model.

**Figure 19:**
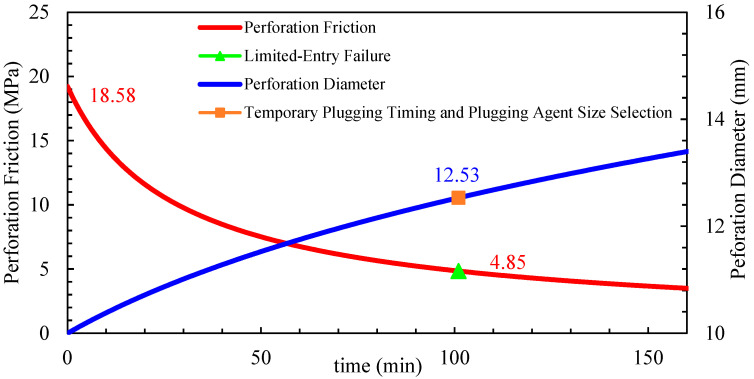
Single-stage 18 perforation holes erosion dynamics prediction chart.

Stage 7: Designed with a pumping rate of 10 m^3^/min, 18 perforations per stage, and 160 kg/m^3^ sand concentrations. The inter-cluster stress difference was 4.85 MPa. Based on the perforation erosion model, the dynamic erosion changes are shown in Figure 19. When perforation friction could no longer balance the inter-cluster stress difference, temporary plugging was initiated at 101 min using 13–15 mm fiber-based diverters.

**Figure 20:**
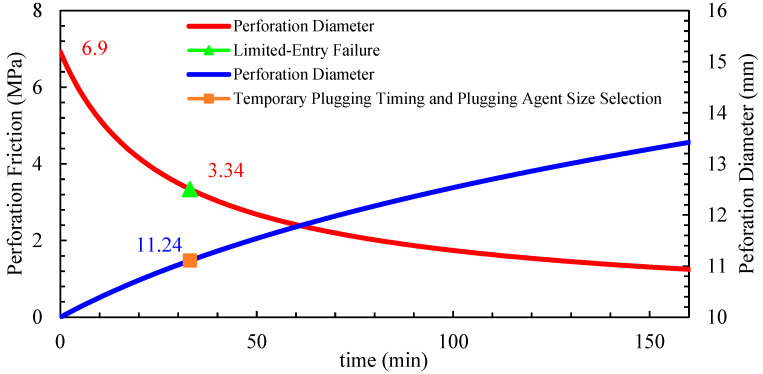
Single-stage 30 perforation holes erosion dynamics prediction chart.

Stage 8: Designed with a pumping rate of 10 m^3^/min, 30 perforations per stage, and 160 kg/m^3^ sand concentrations. The inter-cluster stress difference was 3.34 MPa. Based on the perforation erosion model, the dynamic erosion changes are shown in Figure 20. Temporary plugging was initiated at 33 min using 13–15 mm fiber-based diverters.

**Figure 21:**
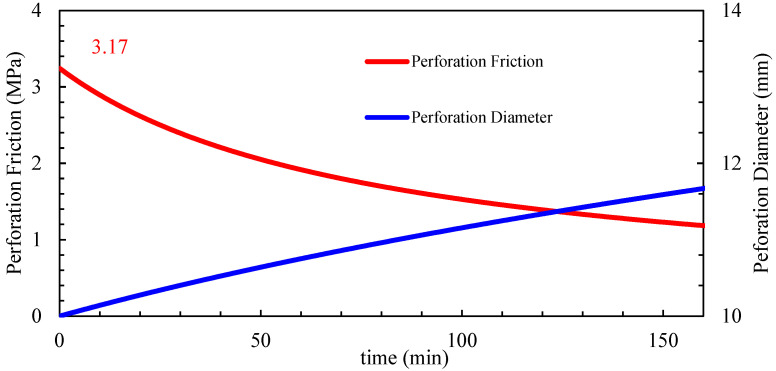
Single-stage 48 perforation holes erosion dynamics prediction chart.

Stage 9: Designed with a pumping rate of 10 m^3^/min, 48 perforations per stage, and 160 kg/m^3^ sand concentrations. The inter-cluster stress difference was 3.8 MPa. Based on the perforation erosion model, the dynamic erosion changes are shown in Figure 21. With 48 perforations, the initial perforation friction was 3.17 MPa, which is insufficient for limited-entry effectiveness. Pre-stage plugging was applied before proppant injection to redirect fluid flow and promote fracture extension.

The limited-entry technique alone is insufficient to address the challenges of low porosity and permeability in the Baikouquan Formation of the Mahu Sag, as well as uneven fracture propagation under heterogeneous formation conditions and large-scale sand fracturing. The integration of temporary plugging optimizes fluid distribution, mitigates perforation erosion, and improves fracture uniformity. This study introduces a “limited entry + temporary plugging” composite process supported by a dynamic perforation erosion model, achieving effective stimulation in Well A. The erosion rate was reduced by 35–50%, fracture uniformity improved to over 80%, and single-well productivity increased by 18–25%. The well achieved a peak daily oil production of 40.55 m^3^/d and a cumulative oil production of 2083.11 m^3^ over 165 days, demonstrating significant reservoir stimulation and enhanced development outcomes.

## 6. Conclusions

Addressing the severe perforation erosion in horizontal well staged fracturing under “high pressure, high rate, and high sand ratio” conditions in the Mahu reservoirs, this study establishes a quantitative perforation erosion model through field experiments, yielding the following key conclusions:

(1) Field experiments indicate that pumping rate, proppant size, concentration, and fluid viscosity are the primary controlling factors of erosion. Ceramic proppants and low-viscosity fluids exacerbate erosion. High-viscosity sand-carrying fluids (≥50 mPa·s) and high-mesh quartz sand (40/70 mesh) are recommended to mitigate erosion risks.

(2) The improved Long model reveals a two-stage erosion process: a sharp pressure drop (>30%) during the roundness erosion stage, followed by a gradual pressure decline (<5%) during the diameter erosion stage, ultimately forming a trumpet-shaped perforation with an inner diameter exceeding twice the outer diameter.

(3) A “limited entry + temporary plugging” dynamic control composite process is proposed. By determining the critical perforation friction for limited-entry failure based on inter-cluster stress differences, the timing and size selection criteria for temporary diverters are optimized, achieving balanced fracture propagation. Field applications resulted in productivity increases of 18–25%.

## Figures and Tables

**Table 1:** Experimental Plan.

Experiment	Flow Rate (m³/min)	Proppant Concentration (kg/m³)	Proppant Size and Type	Erosion Coefficient (*α*)	Erosion Coefficient (*β*)
1	0.9 (High Viscosity)	90	40/70 Mesh Quartz Sand	1.17 × 10^−13^	9.01 × 10^−11^
2	0.9	90	40/70 Mesh Ceramic	1.34 × 10^−13^	5.66 × 10^−11^
3	0.9	90	40/70 Mesh Quartz Sand	3.96 × 10^−13^	1.43 × 10^−10^
		160		4.51 × 10^−13^	1.60 × 10^−10^
		240		3.80 × 10^−13^	1.68 × 10^−10^
4	0.9	160	20/40 Mesh Quartz Sand	3.10 × 10^−13^	1.16 × 10^−10^

**Table 2:** Perforation Scheme for Well A.

Perforations per Cluster	Clusters per Stage	Perforations per Stage	Fracturing Stages
3	6	18	Stages 7, 10, 13
5	6	30	Stages 8, 11, 14
8	6	48	Stages 9, 12, 15

## Data Availability

The original contributions presented in the study are included in the article, further inquiries can be directed to the corresponding author.
